# Rethinking curriculum: A pandemic opportunity for re-engagement with the Arts?

**DOI:** 10.1007/s41297-022-00170-y

**Published:** 2022-08-01

**Authors:** Margaret Cunningham, Robyn Gibson

**Affiliations:** Sydney School of Education and Social Work, Faculty of Arts and Social Sciences, The University of Sydney, Sydney, NSW Australia

**Keywords:** Education policy makers, Curriculum design, Growth mindset, Pandemic opportunity, The Arts

## Abstract

Article 26 of the Universal Declaration of Human Rights endorses the goal of education for all children. Inequalities of access and equity, however, are apparent in both the developed and developing world, which indicates that this goal remains aspirational. The rupture of education during the COVID-19 pandemic has amplified these existing inequalities in education systems worldwide. Throughout the pandemic, teachers have modelled a positive mindset as they pivoted from the physical classroom to online learning. This paper will suggest that the current pandemic may indeed offer education policy makers an opportunity to re-think curriculum design and, with a growth mindset, re-engage with the Arts as an equal key learning area in the curriculum. It is to the Arts that schools and communities have turned for joyful and multi-layered support during the pandemic, an underlying belief in the importance of the Arts for the wellbeing and cognitive development of the child. This belief, supported by a robust body of evidence amassed internationally over many decades, is central to the authors’ contention that a more inclusive and comprehensive engagement with the Arts would facilitate curriculum reform, such as that sought by the New South Wales (NSW) government in Australia. Furthermore, we contend that the COVID-19 pandemic may be the necessary catalyst to activate such welcome reform. We anticipate our analysis and its findings to first be relevant for the state of NSW, then beyond, to resonate nationally and internationally.

## Introduction

The year 1948 was a time of remarkable global change. At the United Nations (UN), the goal of education for all children was endorsed in Article 26 of the Universal Declaration of Human Rights. Of the thirty articles contained in the Declaration, that relating to education received near universal support (Reimers, 2021). The UN understood from its outset the fundamental importance of an educated global community. In less than a month, that priority was evidenced in the creation of the United Nations Educational, Scientific and Cultural Organization (UNESCO). Reimers (2021) believes that “the impact of this global education movement is nothing short of remarkable” adding that it is “the most significant silent revolution experienced by humanity” (p. 10). With the value of education thus emphasised, it is understandable that when the COVID-19 virus was declared a pandemic by the World Health Organization (WHO) on 11 March 2020, an immediate major concern was how best to maintain a continuity of education for all children amidst this devastating global disruption. With this foregrounded, the purpose of this paper is to identify and discuss the interplay of relevant research evidence and government policies that may impact this concern.

Decades of faltering educational policies have been exposed during the COVID-19 pandemic (Schleicher, 2020). As a consequence, existing inequalities and declining excellence in education have been amplified in many countries. While the “catch-up” discourse prevailing among numerous education authorities and policy makers is understandable, the re-building of the post-pandemic global education systems demands an added infusion of imagination and creative thinking. Future-focused educators, despite increasing calls to address these issues, have often been thwarted by a stalled adherence to the familiarity of the traditional educational model; termed a “grammar of schooling” by Tyack and Tobin (1994, p. 454). Regardless of the continuity of this paradigm across the generations, Tyack and Tobin (1994) maintain that change, gradually introduced, is possible once a new sense of the common good is established following issues-related public discourse. The authors consider the complexities of such a paradigm shift in the thinking of policy makers, educators, and the community by focusing on a specific example of proposed curriculum reform in New South Wales (NSW), the most populous state in Australia (AU). The aim of the proposed curriculum reform — how best to prepare students for learning and living in the twenty-first century world of continual technological change and uncertainty — is shared by UNESCO (2021) and many countries across the globe including the UK, the US, Singapore, New Zealand (NZ), and Finland. Key issues of concern — equality of access to and inclusion in a broadened future-focused education system — have relevance more widely both within Australia and internationally.

## Background

Recently, a review of the New South Wales (NSW) Kindergarten to Year 12 (K-12) curriculum was conducted by the NSW Education Standards Authority (NESA) “to ensure that the NSW education system is properly preparing students for the challenges and opportunities of the twenty-first century” (NESA, 2020a, p.v).

This was in fact the second major review of the NSW K-12 curriculum in three decades. This, and the earlier review preceding the 1989 Carrick Report, shared a common aim and reached the same conclusion: reform of the NSW curriculum is imperative. In the intervening and relatively short number of years, it is the context in which learning takes place that has changed for many students. Today, an increasing number of routine cognitive and manual occupations are performed by technology. The resulting decline in such employment has been matched by an ever-increasing global demand for a flexible workforce capable of critical and creative thinking, and in possession of effective communication and collaboration skills (Jefferson & Anderson, 2017; Trilling & Fadel, 2009). The challenging and now irreversible changes in twenty-first century society, the result of increasing globalisation, and accelerating transformative advances in technology, now demand an advance on the reproductive pedagogical process of past decades. “As the days of the twenty-first century slip away … there is little time to waste to ensure that the current generation is prepared to lead and succeed” (Soulé & Warrick, 2015, p.185). Hence the advice of the 2020 NESA Report, *Nurturing wonder and igniting passion: Designs for a new curriculum*, a reformed and future focussed NSW Curriculum is now required to provide students with the knowledge and skills most needed to thrive at school and beyond in the twenty-first century.

## Broadening the curriculum

Importantly, the above report reveals a welcome mitigation of the tension apparent between stakeholders and the reviewing bodies in the previous Carrick Report (1989) and that of the NSW Ministry of Education and Youth affairs (Excellence and Equity, 1988), which culminated in the 1990 Education Reform Act. Tellingly, Braithwaite (1992) describes the reforms endorsed at that time as a politicised “blitzkrieg model of curriculum change” (p. 51), lacking a collegial approach. Thus, mindful of earlier controversy over inadequate consultation, an initial key design feature is in response to teachers’ advice concerning the negative impact of an overcrowded curriculum: the incoming curriculum will see its breadth reduced to allow for in-depth learning and understanding of essential concepts and knowledge. Interestingly, a second key feature — the integration of knowledge and skills — was perceptively foreshadowed in 1989 by Sir John Carrick. The transmission of content alone Carrick no longer considered sufficient arguing for “the development in students of initiative, creativity, flexibility, problem solving and the ability to work with others” (Carrick, 1989, p.25), enduring abilities now globally labelled as twenty-first century skills (Jefferson & Anderson, 2017). Clearly, the above-mentioned design features — depth of learning and knowledge application — are highly relevant in this time of ever-expanding knowledge and continuous technological change. The key to their facilitation, however, lies with a third aim of the new curriculum: the fostering of student engagement, and joy — as opposed to apathy or indifference leading to a “crushing boredom” in learning (Johnson, 2020, p. 20). Student engagement, identified as a significant precursor to student learning (Hattie, 2012; Zyngier, 2007), is a complex mix of behavioural, emotional, and cognitive elements (Pedler et al, 2020). There is strong evidence that learning and the emotions are closely intertwined (Darling-Hammond & Hyler, 2020; Hascher, 2010) yet, according to Johnson (2020), there is surprisingly little research on the specific concept of “joy.” Building on the work of Johnson, (2020), Van Cappellen (2020) suggests that “joy connects us to our core identity … It is the emotion that makes life worth living in the moment” (p.40). Related is the experience of flow, a concept developed by Mihaly Csikzentimihalyi (1975). In flow, whether athlete, artist, chess player, or student, the experience is the same; the intense concentration is like breathing, unnoticed. “There’s a joyousness to it. That’s when you’re happiest or that’s when you’re most you or that’s when you feel your best” (Spiegel quoted in Tough, 2012, p.137). Perhaps, it is that intrinsically rewarding experience of flow that curriculum reformers have in mind when advocating for student engagement and joy in learning?

## Pedagogical change

The above outlined features, “the biggest shake up of the education system in thirty years” (NSW government, 2020), indicate a clear desire to overturn long-held assumptions of what learning should look like. That plea for education reform, described by Levin (1998) as a recurring refrain, has been variously expressed by others: a case of “transformative learning” (Stoll, 2020, p. 424), as “seismic shifts in the education landscape” (Sabol, 2013, p. 33), or as an “unlearning of old teaching habits” (McWilliam, 2008, p. 268)). Elaborating on her concept of unlearning, McWilliam (2008) engagingly describes the evolving pedagogical shift in education. “Teachers have un-learned the role of *Sage-on-the-stage*” (p.265), next shifting to the learner focused role of *Guide-on-the-side* and, on reflection, finally settling at the midpoint of the teacher-learner continuum with the role of the teacher as *Meddler-in-the-middle*. Significantly, it is that latter role, which is the salient feature underpinning the realisation of the current reform goals.

There is general national and international agreement that the teacher-learner relationship is crucial in enabling success in learning (for example, Arnold, 2005; Darling-Hammond & Hyler, 2020; Hattie, 2012; Kosnik & Beck, 2009; Lucas & Spencer, 2017; Zyngier, 2007). Respectful dialogic collaboration coupled with motivating teacher enthusiasm are just two aspects of a positive relationship. Therefore, as Hattie (2012) and Zyngier (2007) suggest teachers should be wary of the deficit discourse in which students are responsible for engagement, or not. The key to such engagement and joy in learning rests with the pedagogical process that empowers the student “with a belief that what they do will make a difference to their lives” (Zyngier, 2007, p.73) at school and in the future. To this end, we now consider what perceived failure in education impelled the present revolution in curriculum policy discourse.

### The catalyst

The first thing to note is that NSW is not alone in its demand for curriculum reform. It is an international phenomenon in industrialised countries that has been likened to the spread of disease in an epidemic (Levin, 1998). Levin considered his analogy of a “policy epidemic” to “be a useful heuristic” (p. 138). “New agents of disease tend to spread rapidly as they find the hosts that are least resistant. So it is with policy change in education—new ideas move around quite quickly, but their adoption may depend on the need any given government sees itself having” (p. 138). An apposite analogy applicable to NSW curriculum reform during an evolving pandemic.

Two decades earlier, a global benchmarking instrument, *Knowledge and skills for life*, was launched by the Organisation for Economic Co-operation and Development (OECD) — the triennial Programme for International Student Assessment (PISA) of reading, mathematical and scientific literacies. Being an active member nation of the OECD since joining one decade after its inception in 1961, it is not surprising that Australia chose to participate. In contrast to many other assessments, PISA “based on a dynamic model of lifelong learning” (OECD, 2000, p.14), does not simply assess a student’s capacity to recall facts. Instead, it also examines a student’s ability to reflect and apply that factual knowledge to new situations and problems. In other words, PISA assesses a student’s higher order thinking skills, not a curriculum.

Results for Australian 15-year -old students in 2000 were of a high standard in comparison to most of the participating thirty-two countries. In reading literacy, Australia ranked 4th behind Finland, Canada, and New Zealand. In mathematical literacy, Australia was placed an equal 5th with Canada behind Japan, Korea, NZ, and Finland and an equal 7th with NZ in scientific literacy behind Korea, Japan, Finland, the UK, and Canada (OECD, 2000). A benchmark had been set. Inaugural success, however, has been disrupted; levels of performance have declined and Australia has now slipped in international rankings. Of equal interest, is a comparison of Australia’s intranational results in the 2018 PISA, which revealed marked differences in performance between the states. Overall results for Victoria, for example, saw no significant decline, whereas a significant decline in achievement was recorded in the other states. To illustrate, mathematics results for South Australia (SA), Western Australia, (WA), Tasmania (TAS), and NSW, all recorded a significant decline. The latter alone are disturbing statistics. For NSW, however, concern has been compounded with PISA 2018 results for reading and scientific literacy being the lowest in the nation. In reading, the focus of PISA 2018, Australia’s trajectory is graphed as “steadily negative” (Schleicher, 2018, p. 11). Might the diminishing capabilities of Australian students, as evidenced in the above PISA 2018 results, be the ‘scandalising’ (Steiner-Khamsi & Waldow, 2018, p. 560) catalyst for the recent major review of curriculum in NSW? After all, as the report acknowledges, teachers have been making robust calls for change for quite some time. Interestingly, the statistics also suggest that the catalyst for reform may be operational on two levels: internationally as is generally understood — a “Sputnik shock” for the twenty-first century — and nationally, as signalled by a comparison of results attained by students in Victoria and NSW. The question is how might a transformation of learning be implemented?

## Implementation

The title of the report — *Nurturing wonder and igniting passion: Designs for a new curriculum* — and its long-term vision to nurture wonder and ignite passion in all learners is an exciting prospect to contemplate. Stakeholder demands have been acknowledged: flexible and in-depth learning from teachers, learning with real-world application from students and parents, and from employers a demand for the social skills of collaboration and communication alongside foundational literacy and numeracy skills. The challenge now rests with the transformative process of implementation. Encouragingly, the first step has been achieved — the acknowledgement that the intertwining of knowledge and skills is crucial to best prepare learners for success at school and beyond. This notion, fundamental to the potential transformation of the curriculum, is illustrated by the following brief excerpt: “Rather than being taught or assessed separately from subjects, such skills are incorporated into new syllabuses and are seen as an integral part of developing competence in each subject” (NESA, 2020b. *Executive summary*, n.p.). Furthermore, and again in response to substantial research evidence, an ensemble of social and emotional influences is also stressed, less tangible but equally underpinning successful learning (Farah, 2017; Goleman, 1995; Smilkstein, 2011). To thrive, for instance, a supportive and safe learning environment at school contributes to the wellbeing of the student, which in turn impacts on a student’s motivation to learn and fosters their feeling of belonging, the sense of being part of a “community of learners” (Arnold, 2005; Costa, 2008; Eisner, 2002; Gibson & Ewing, 2020; Hascher, 2010; Jefferson & Anderson, 2017; Rogoff, 1994; UNICEF Innocenti Report, 2020; to name a few). In text, the design features of the reformed curriculum are encouraging aspirations. In practice, implementing to “nurture wonder and ignite passion” may be more problematic.

Focused predominantly on the basic skills of literacy and numeracy, the proposed NSW curriculum reforms echo a familiar refrain. Irrespective of which political party may be in power, through the past decades and now into the third decade of the twenty-first century, it seems that a “back-to-basics” cry remains the persistent reform solution to perceived educational shortcomings. Is it possible for more-of-the same to ignite passion and joy of learning?

A reformed curriculum, nevertheless, suggests an improved curriculum. Also implied is that such reform is occurring “for the better.” Yet, as Mockler and Groundwater-Smith (2018) suggest, the use of the words “reform” and “improvement” is pernicious, “disguising meaning and driving practitioners to conformity and compliance” (p. 2). Yet again, it might also include the notion, as Horth and Mitchell (2017) state, that innovative reform ideas are “fragile things … easy to get spooked by … and can seem ambiguous and risky” (p.1). Thus, the very creativity that is required for reform is sabotaged. Reassuringly, however, it is elsewhere affirmed in the report that “the new curriculum does not propose that children spend inordinate amounts of time on reading and mathematics to the exclusion of other aspects of learning, including physical activity, play, music and art” (NESA, 2020b. *Executive summary*, n.p.). Heartening words certainly, but nevertheless hinting at an unfounded disconnection between the development of the whole child and cognition. Numerous studies evidence the importance of play and physical activity for the mental and physical health of children (Bateson & Martin, 2013; Sahlberg & Doyle, 2019), for cognition (Brown & Vaughan, 2010; Kang, 2020; McWilliam, 2008), and for social and emotional development (Chmelynski, 2006; Sahlberg & Doyle, 2019). However, play and physical activity, often trivialised, are in decline both in and out of school. More sedentary “desk time” to ensure high scores in standardised testing “may have a certain naive logic but it happens to be dead wrong. … Play is cognitively challenging. … It thrives on complexity, uncertainty, and possibility, which makes play just about the perfect preparation for life in the twenty-first century” (Marano, quoted in Chmelynski, 2006, p.13). In play and physical activity, as Sahlberg and Doyle (2019) and others assert, children have an opportunity to fuel their curiosity and with imagination create new ideas. Imagination is a core capability to ponder possibilities (Beghetto & Schuh, 2020; Craft, 2015; Eisner, 2002) and to ask, as Craft posits, “what if?” (2015, p. 153). It is crucial that their position is secured in the proposed transformation of the school curriculum in NSW. Likewise, there is unequivocal research evidence that it is through the lens of quality arts experiences that a curriculum may potentially be transformed (Ewing, 2020). In turn, engagement in quality arts programs fosters the development of skills and thinking dispositions that students require for learning and life in the twenty-first century.

A disposition, broadly speaking, is an individual’s idiosyncratic response to a challenge and is variously described as a habit of mind (Costa & Kallick, 2008; Hogan & Winner, 2019). Thinking dispositions go beyond the capacity to recall factual knowledge (Perkins et al, 1993). Instead, as Tishman et al, (1993) explain: “A good thinker possesses … abiding tendencies to explore, inquire, seek clarification, take intellectual risks, and think critically and imaginatively” (p.147), in turn to be activated by a trio of abilities, sensitivities, and inclinations. Expanding on this theme, Winner and Hetland (2008) compellingly argue that in this world of constant and rapid change, the Arts teach students “vital modes of seeing, imagining, inventing, and thinking” (p.31), which will best equip them to come up with novel solutions to present and future problems. Imagination has offered release from an out-of-date curriculum. In the twenty-first century, to be the “mind-altering device,” as a curriculum has been described by Elliot Eisner (2002, p.13), the new NSW curriculum reforms might best explore its possibilities through the lens of the Arts.

## The Arts in education

The creative arts **(**dance, drama, music and visual arts), one of eight key learning areas (KLAs) in the NSW curriculum, are frequently marginalised transnationally by government policy and their position in the curriculum is often contested. Writing from an English perspective, Heaton and Hickman (2020) agree that their cognitive value is also disregarded. In spite of such obstructions, research of and support for the Arts in education has persisted internationally (Caldwell et al, 2021; Caldwell & Vaughan, 2012; Catterall, 2012; Dewey, 1934; Eisner, 2002; Fiske, 1999; Kisida & Bowen, 2019; Lum & Wagner, 2019; Winner & Hetland, 2008) and nationally (Ewing, 2020; Fleming et al., 2016; Gibson & Ewing, 2020). Separately, a variety of inquiries has investigated the place and state of the Arts in education across the nation.

Prior to the earlier mentioned Carrick Report (1989), such an inquiry was initiated in 1975 with nine reports being issued during 1976 to 1977. These reports are of immense significance as they are “the first documents reflecting a national concern and commitment to the Arts as a recognised and important part of the curricula of schools” (Lett, 1981, p.3). Subsequent inquiries into arts education within Australia include the following:

- 1980 Lett, W.R. A *survey and report on the needs and priorities for research in arts education in Australia.*
- 1995 Coulter, J. *Arts education: Report by the senate environment, recreation, communications and the arts references committee.*
- 2008 Davis, D. *First we see: The national review of visual education.*
- 2009, 2013, 2016, 2020 Australia Council for the Arts*: National arts participation surveys.*
- 2010 Ewing, R. *The arts and Australian education: Realising potential.*
- 2015 *The Australian curriculum: The arts.*

## The Arts: political status

With such continued attention demonstrating an awareness of the relevance of the Arts in education, it is puzzling to find the role of NSW Minister for the Arts diminished. Launched in 1971, a Minister for Cultural Activities was appointed to manage and support the Arts. In succeeding years, the Arts were incorporated in various ministries and departments: The Ministry of Culture, Sport and Recreation (1975), Ministry of the Arts (1988), and contained within the Police Justice Department (2014). Today in NSW, the Arts are the responsibility of the Minister for the Public Service and Employee Relations, Aboriginal Affairs, and the Arts*.* Federally, the Arts are similarly managed under an umbrella of ministerial responsibilities: Minister for Communications, Urban Infrastructure, Cities, and the Arts.

Tacked on to a loosely related group of responsibilities, the political perception of the Arts appears to be at odds with that of the community as revealed in the Australia Council for the Arts report, *Creating our future* (2020)*.* In survey responses, 75% of NSW citizens stated that they considered the Arts to be an important part of education, had a positive impact on wellbeing, and contributed to the shaping of the Australian identity.

## The Arts: curriculum

The disparate attitudes towards the Arts are reflected in the proposed new NSW curriculum. As a curriculum is a joint creation of the community and politicians alike, a contested view may be inherent in its construction. While not stated, the notion of a “curriculum hierarchy” (Bleazby, 2015) — the idea that some school subjects are more valuable than others — is suggested. The apparent contested attitude towards the Arts is significant, as “the school curriculum is a cultural construction” (Kennedy, 2019, p.121), in which the knowledge, skills, and beliefs of a community are preserved. Importantly, principles of democracy are also implied in Kennedy’s expanded argument: “Curriculum is about the collective—what is best for everyone” (p. 123). Also implicit is the principle of social inclusion, a concept measured across the PISA 2018 participating countries according to the degree of segregation across schools and resources allocated. The results, published in an “Index of social inclusion,” were striking: Australia ranked lowest (Schleicher, 2018, p. 23).

The states and territories within Australia develop an individual curriculum, a consequence of which is an idiosyncratic expression of priorities. The NSW curriculum, for example, traditionally emphasises academic content, rigour, and competitive assessment. Pride in its rigour, critics argue, is a mindset that typically favours the privileged, thus weakening the inclusion related concept of equality (Hughes, 2019; Yates et al, 2011). The curriculum “shake up” — as the review was described by the NSW government at its launch in 2018 — implies a potentially substantial shift in thinking. To illustrate a possible start, the report declares that rote learning of factual knowledge is to be kept to a minimum to “enhance engagement and enjoyment of learning” (NESA, 2020b. *Executive summary*, np), thereby also suggesting the importance of the learner’s social and emotional wellbeing. Again, the title — *NSW curriculum review report: Nurturing wonder and igniting passion* — is also suggestive of significant change: a shift towards more flexible and creative thinking, a counter to present rigidity, and standardised testing. Furthermore, as stated in the long-term vision, the goal for the reformed curriculum is “to nurture wonder, ignite passion and provide every young person with knowledge, skills and attributes that will help prepare them for a lifetime of learning, meaningful adult employment and effective citizenship” (NESA, 2020b. *Executive summary*, np), a welcome and encouraging vision for the future. A vision, however, that could equally be shared by a curriculum of the Arts.

A pedagogical tension is thus suggested. Curiously, publication during the COVID-19 pandemic may have delivered the unexpected outcome of nudging curriculum reform further towards the Arts and curriculum transformation. The revolutionary necessity of online learning, for instance, is now accepted and teachers resiliently demonstrate flexibility. Might we have reached a “tipping point” — a concept investigated by Gladwell in his book of the same name (2000) — where the Arts have a valued place in the twenty-first century model of education? Diminished success in the 2018 PISA disrupted traditional educational thinking, might also the disruption of the pandemic be the catalyst for acknowledgement of the decades of research evidence highlighting the value of the Arts in education? Schools and their communities certainly have. It is to the Arts that schools and communities have turned for joyful multi-layered support during the pandemic: online visual and musical montages, for example, connecting students and teachers, locked-down art museums creatively opening their online doors to the global world. Arguably, the foundation for transformation has been laid. Acknowledging an urgent need for curriculum reform — that “shake up” demanded of the curriculum review — indicates an essential shift in the mindset of policy makers. That “crack” in a habit of thinking, where, as the song says, “a crack where the light gets in” (Cohen, 1992), to invoke an apt arts analogy. The opening is now there to consider how a pedagogical approach through the lens of the Arts may provide the support necessary for achieving goals expressed in the NESA 2020 Report.

## Growth mindset in teaching

Intentionally changing a curriculum is easier if one knows by how much and why (Duckworth & Gross, 2020). Although visionary, the parameters of the new NSW curriculum are neither defined nor is a mechanism of implementation offered. Instead, although not stated, a growth mindset of teacher and school underscores the possibility of achieving curriculum transformation. In this instance, a revisioning of pedagogy through the lens of the Arts provides a possible mechanism for the desired transformation.

As welcome as an improved new curriculum may be, publication and its rhetoric do not equal transformation (Hattie, 2012); “curriculum goals don’t teach themselves” (Reimers, 2021, p. 17). In the twenty-first century, a consequence of global anxiety related to the high stakes testing environment has been a frequent parallel narrowing of curriculum content and marginalisation of the Arts (Berliner, 2011). Over the past several decades, as earlier stated, a growing body of national and international research evidence repeatedly underscores the fundamental cognitive, affective and social importance of the Arts in education. It seems, then, that not enough attention is being paid to the research. On further reflection, an additional element is highlighted: the difficulty of breaking away from the comfort and familiarity of habituated thinking (Duckworth & Gross, 2020; Latham & Ewing, 2018). Yet, when facing the challenges created by the unexpected disruption of the COVID-19 pandemic, teachers across the world revealed a growth mindset in their willingness to do so. An immediate upgrading of digital proficiency, for instance, was required by many teachers as they navigated a path for their students from the traditional classroom to online learning. With numerous inequities now exposed in global school systems (Schleicher, 2020), many educationists are calling “for an overhaul of an industrial model of schooling and … that there is no way back to the old model of school education” (Sahlberg, 2020, p.363). Offering similar advice, Reimers and Schleicher (2020) stress the enormous potential of the pandemic crisis to fuel innovation within education systems. Nudged, already, in the direction of change necessitated by pandemic disruption, the authors further argue that the pandemic offers a revitalising catalyst for curriculum and pedagogical change by engaging in educational reform through the lens of the Arts. The Arts, for too long placed “in a realm of their own, disconnected from other modes of experiencing” (Dewey, 1934, p.9), offer educators the means to nurture wonder and ignite passion in their students in NSW and beyond.

## Learning through the lens of the Arts

A sense of uncertainty permeates all aspects of society during the present deep cross-national pandemic crisis. Uncertainty frequently has a negative connotation suggesting a lack of control and predictability, or a sense of potential peril. Yet, as scholars (Beghetto, 2020; Craft, 2000, 2015; Glaveanu, 2018) observe, there is a positive aspect to the concept of uncertainty; uncertainty may be the catalyst for promoting new ways of thinking and action to solve a problem. In this case, educators around the world are uncertain of the impact of school closures on student learning and wellbeing now and into the future. The role of uncertainty has been variously described as “possibility thinking,” a term coined by Anna Craft (2000), “a gateway to the possible” (Beghetto, 2020, p.1**)**, or as earlier described in this paper, as “a crack where the light gets in” (Cohen, 1992). Whichever iteration of the positive role of uncertainty is preferred, all indicate a catalytic action for change in thought and action.

To transform education from the “past normal” also requires imagination and reflection. Many educationalists have been doing so over the past few decades as they consider the optimum ways to prepare students for school and life in this century of massive technological and societal change (Dede, 2010; Fiske, 1999; Jefferson & Anderson, 2017; Trilling & Fadel, 2009; Warner, 2006). “Our definition of education must be broadened” (Florida, 2014, p.390) beyond the foundational basics of literacy — read, write, speak, and listen effectively — and numeracy. To that end, several international frameworks were established to identify twenty-first century competencies deemed essential for optimum learning, which Voogt and Roblin (2012) found “to be largely consistent in terms of what twenty-first century competences are” (p. 306). There was strong agreement, for instance, on the need for skill in the areas of communication and collaboration, creativity, and critical thinking. One such example is the US-based Partnership for 21st Century Learning (P21), which distilled eighteen essential skills into the four interdependent competencies of critical thinking, communication, collaboration, and creativity, generally referred to as the “4Cs”, timeless but now foregrounded skills specifically mentioned in the new NSW Curriculum “to be developed in parallel with [a student’s] advancing knowledge and understanding of each subject” (NESA, 2020b. *Executive summary*, n.p). These are cross-disciplinary skills which may be nurtured through quality arts processes and experiences, and integrated across all key learning areas (Ewing, 2020). Cognitive and psychological benefits are numerous and include, for example, critical and creative thinking (Kisida & Bowen, 2019; Wilson et al, 2021; Winner et al, 2013) self-efficacy and creativity (Gibson & Ewing, 2020; Mansour et al, 2016); interpersonal communication and collaboration skills (Burton et al., 2000; Thompson & Tawell, 2017); empathy (de Eca et al, 2017; Riddett-Moore, 2009); flexible thinking, resilience, and perseverance (Eisner, 2002; Hetland et al, 2013), and increased student and parent school engagement (Bamford, 2006; Caldwell & Vaughan, 2012).

## The pandemic

The advice of researchers Perales and Arostegui (2021) is to go beyond the neoliberal agenda “to take advantage of this historical moment to transform education toward a more humanistic approach … offer[ing] a well-rounded education to new generations” (p.1). Likewise, research by Gianmarco et al. (2020) found social and emotional “human skills” to be of the most value in learning and the workplace. OECD research into the responses of schools and countries to the pandemic — *Schooling disrupted, schooling rethought: How the COVID-19 pandemic is changing education* (Reimers & Schleicher, 2020) — reveal, however, a familiar tension: some have prioritised core curriculum content essential for testing, others “consider that the crisis has shown the need to foster a wider range of cognitive, social and emotional competencies, and focus on student well-being” (p. 6). What is striking is the gap in responses between “government representatives” (p.6) and those of teachers: the former tend to focus on academic learning, the latter emphasises the need to “bolster student engagement” (p.6). Teachers are agents of change; John Hattie (2012) informed us a decade ago. Like the “growth mindset” of Dweck (2006), Hattie (2012) considers the “mind frames” of teachers and school executive to be the crucial factor in activating change.

## Conclusion — a growth mindset for change

“Real change often takes place in deep crises” write Reimers and Schleicher in their OECD report (2020, p.9). While many have “high hopes” for an education transformation, Sahlberg (among others) strongly argues that “there is little chance schools will change as a consequence of this pandemic” (2020, p. 359). However, an added caveat, with a hint of positivity, concludes that a reimagining of education c*ould* occur with “bold and brave shifts in mindset” (p.359) of teachers and decision makers. The COVID-19 pandemic has amplified existing weaknesses and inequalities in society and education; the notion of a democratic education has been diminished (Adams & Owens, 2016). For Australia, ranked lowest for inclusion in the 2018 PISA, this is a significant concern. Research evidence, however, indicates that the “playing field” may be levelled in part by offering all students — privileged and disadvantaged — equal access to quality arts programs, with the latter afforded the most benefit (Bamford, 2006; Catterall, 2012; Fiske, 1999; Gibson & Ewing, 2020). Throughout the pandemic, (most) government officials and citizens have respected, valued, and heeded the advice of the medical experts. Likewise, now is an optimum time for government and education policy makers to reflect on the compelling body of research evidencing the valuable role of the Arts in learning for *all* school students. Singapore, globally admired for its high-performing education system, has done so. Mindful of research findings and demonstrating a growth mindset, Singapore authorities, beginning in 2016, broadened their narrow assessment oriented academic approach to one “where educators create environments which inspire students to explore, showcase their creativity, build confidence, and own their learning”. *Nurturing wonder and igniting passion*: *Designs for a new curriculum*, the title of the NSW Curriculum Review Report, similarly conveys the intention for student and teacher alike to share a sense of joy and excitement in learning. We contend that this is possible, despite evidence of paradoxical reasoning in the report.

Developments in the science of learning are acknowledged yet a stalled reliance on the tools of the less complex past — “the basics” and competitive standardised testing — are also evident. Similarly perplexing is the proposed significant depreciation of arts engagement for young people when their value is recognised by the specific KLA of the *Creative Arts*. Such indecisive reasoning, as frustrating as it is, is also encouraging. Implied is a sense of a changing mindset as policy makers grapple with the unspoken challenge of rupturing habituated thinking, a sense of motion towards a tipping point.

The above demonstrates the complexity of re-thinking curriculum. Past practices, we argue, demand amendment but paradoxically, again, the past presents a constructive solution to facilitate political re-engagement with the Arts: return the 1971 NSW *Minister for Cultural Activities* or even recreate the *Ministry of the Arts* as in 1988. In the present, a broader understanding of the science of learning demands acknowledgement and recognition of the compelling body of evidence demonstrating the cognitive, affective, and social benefits of the Arts. With a growth mindset, activated in education stakeholders by the “deep crisis” catalyst of the COVID-19 pandemic, the presently marginalised *Creative Arts* may be re-established as an effective KLA in the NSW curriculum. Such a result would offer a potential reward of the deeper learning now required to meet the present and future multifaceted needs of learners (Darling-Hammond et al, 2020; Kisida & Bowen, 2019). It would also align NSW with Australia’s close neighbour NZ, where the future-focused Principals’ Federation declares that the Arts will be essential in a post-coronavirus world (Christian, 2020; Rush, 2020).
